# Concrete Damage Identification and Localization for Structural Health Monitoring Based on Piezoelectric Sensors

**DOI:** 10.3390/s25082532

**Published:** 2025-04-17

**Authors:** Hongjie Li, Bo Di, Yu Zheng, Hongwei Ma, Xiaomiao Huang, Hekun Wu, Jize Zhang

**Affiliations:** 1 School of Environment and Civil Engineering, Dongguan University of Technology, Dongguan 523808, China; 2 Guangdong Provincial Key Laboratory of Intelligent Disaster Prevention and Emergency Technologies for Urban Lifeline Engineering, Dongguan 523808, China; 3 China Harbour Engineering Company Ltd., Macao 999078, China; 4 China Construction Fifth Engineering Division Company Ltd., Changsha 410004, China; 5 Department of Civil and Environmental Engineering, Hong Kong University of Science and Technology, Hong Kong 999077, China

**Keywords:** concrete damage detection, localization, structural health monitoring, piezoelectric sensors, non-destructive testing

## Abstract

In recent years, effective methods for concrete damage identification and localization have become crucial in the field of structural health monitoring (SHM). This study proposes an approach utilizing piezoelectric sensors to detect and localize damage in concrete structures. The method involves using a network of piezoelectric ceramic sensors to actively excite and receive stress waves within the concrete. By analyzing the differences in wave propagation between healthy and damaged states, internal damage can be identified and localized. The collected data are processed using advanced signal processing techniques, including wavelet analysis and pattern recognition algorithms, to accurately identify the damage’s location and severity. Experimental results demonstrate the high precision of this method. Compared to traditional techniques, this approach offers significant advantages, including faster detection, non-destructive testing, and real-time monitoring. In conclusion, the use of piezoelectric sensors for damage detection and localization provides a promising solution for enhancing the safety and longevity of concrete structures, offering a reliable tool for structural health monitoring in civil engineering applications.

## 1. Introduction

Concrete is one of the most widely used construction materials globally, owing to its strength, versatility, and cost-effectiveness. However, concrete structures, such as bridges, buildings, and highways, are susceptible to damage from various sources, including mechanical loads, environmental exposure, and material aging. If such damage remains undetected, it can lead to catastrophic structural failures, posing significant safety risks and incurring high repair costs. For example, the collapse of the Tacoma Narrows Bridge in 1940 [1] and the recent partial collapse of the Morandi Bridge in Italy in 2018 were partly attributed to undetected structural damage, resulting in tragic loss of life and severe economic consequences [2]. In the case of the Morandi Bridge, corrosion and fatigue damage were present for years before the failure, but routine inspections failed to detect the severity of the damage in time [2]. Therefore, early detection and accurate localization of damage are critical for maintaining the structural integrity and safety of concrete infrastructures. Timely intervention can prevent catastrophic failures, reduce maintenance costs, and extend the service life of the infrastructure.

Currently, concrete damage detection technologies are classified into two main categories: destructive testing and non-destructive testing (NDT). Destructive testing involves sampling operations on the structure, which can affect the overall performance and durability of the concrete. Consequently, destructive testing cannot be performed on a large scale, limiting the scope of detection [3,4,5]. In contrast, NDT methods detect damage without harming the structure. These techniques use various sensors and associated equipment to detect changes in the structure caused by internal damage, enabling the identification, localization, and quantification of the damage severity [4,5,6].

However, traditional NDT methods, such as visual inspections [7], ultrasonic testing [8], and radiography [9], have inherent limitations related to accessibility, cost, and the inability to detect sub-surface or early-stage damage. As a result, there is a growing need for advanced, non-destructive techniques capable of providing real-time, accurate assessments of concrete conditions. Structural health monitoring (SHM) is an advanced extension of NDT technology, involving the deployment of a network of sensors on or inside the structure to collect real-time data. The data are then analyzed to assess the health status of the structure [10,11]. SHM provides essential information for evaluating the health of a structure without compromising its overall performance, offering early warnings when damage occurs, and enabling timely interventions to reduce or even prevent accidents, thereby minimizing risks to human lives and economic losses [11,12]. In addition to traditional ultrasonic and piezoelectric-based SHM techniques, recent studies have explored microwave-based NDT methods that utilize the dielectric properties of concrete to detect internal anomalies. These methods rely on high-frequency electromagnetic wave propagation and changes in dielectric constant to infer the presence of voids, cracks, or moisture content [13,14,15].

In recent years, piezoelectric sensors have emerged as a promising tool for SHM. These sensors, which convert mechanical stress into electrical signals, offer several advantages, including ease of integration, cost-effectiveness, and the ability to continuously monitor large-scale structures [16,17]. Piezoelectric sensors can detect acoustic emissions generated by internal damage within concrete, providing direct indications of internal cracks, delaminations, or other forms of structural deterioration. Several studies have demonstrated the potential of piezoelectric sensors for SHM applications, particularly for damage detection and localization in concrete structures [18,19,20]. For instance, the Electro-mechanical Impedance method utilizes the high-frequency vibration response of piezoelectric sensors to infer local stiffness changes in the host structure [21]. This technique is highly sensitive to local damage but typically requires very fine mesh sensor placement and precise baseline data. Another widely applied method is Acoustic Emission monitoring, which detects stress waves passively emitted by crack propagation events [22]. It provides real-time monitoring capability and is well-suited for crack detection, but its interpretation depends heavily on source mechanisms and wave path characteristics. Similarly, Gao et al. [23] applied scattering analysis of wavefields to detect delamination and voids in concrete slabs. However, challenges remain in optimizing sensor placement, improving data processing algorithms, and ensuring reliable localization of damage, particularly in complex and large-scale structures [24,25,26].

Despite these advances, several challenges remain. Many existing methods rely heavily on signal frequency-domain analysis or wave mode decomposition, which increases computational complexity and reduces real-time applicability. Others require dense sensor networks or extensive prior knowledge of the structural model to achieve accurate localization, limiting their generalizability to diverse geometries and damage types. Furthermore, in some cases, the identification of internal damage still depends on indirect indicators such as stiffness loss or changes in resonant frequency, which may lack spatial resolution or sensitivity to small-scale defects.

To overcome these limitations, this study proposes a damage identification and localization method based on damage scattering signals and minimal sensor input. The method extracts the first arrival time of the damage-induced scattering wave and constructs ellipses defined by sensor pairs to geometrically infer the damage location—without relying on structural models or dense sensor arrays. This approach not only reduces signal processing complexity but also improves localization efficiency and robustness across varying damage conditions. Moreover, the proposed method is validated through both experimental and finite element simulation data, covering different types and severities of damage. These contributions offer a practical and scalable solution for SHM in concrete infrastructures. The primary work conducted in this study is as follows: In Section 1, the background and significance of the study, including SHM and the application of piezoelectric sensors, are introduced. Section 2 focuses on the damage localization methods, presenting the theory of stress wave propagation in concrete and explaining methods for determining the stress wave speed and acquiring damage scattering signals. In Section 3, experimental results on wave analysis to identify and localize common concrete damage are reported. Different sizes and positions of hole defects are artificially created by reserving holes in the concrete, and Smart Aggregates (SA) are attached to the surface to transmit and receive signals. By analyzing the damage scattering signals, the location of the damage is determined. The results are compared with the actual damage conditions to validate the effectiveness of the damage localization method. In Section 4, the finite element method with wave analysis is used to identify and localize concrete damage. Finite element software simulates the damage and provides data results. These simulation results are compared with experimental results to verify the feasibility of the finite element model and confirm the validity of the evaluation results. The main innovations of this study are as follows: (1) Development of a concrete damage identification and localization method based on damage scattering signals and stress wave speed. (2) This method analyzes the response signals in the time domain, reducing the complexity of data analysis and saving significant analysis time, making the localization of concrete damage more efficient. (3) This method is an analysis-based approach using response signals, which does not rely on structural models, making it more suitable for practical engineering applications.

## 2. Materials and Methods

This section outlines the research methodology used in this study, which focuses on damage localization based on damage scattering signals in concrete. The underlying principle of this method is wave analysis. A specific excitation signal is applied to the actuator, and, due to the inverse piezoelectric effect of the piezoelectric sensors, the actuator generates stress waves in the structure. These stress waves propagate through the medium and are detected by the sensors [27]. By comparing the response signals collected under healthy and damaged conditions, the differences between the signals are obtained, representing the changes induced by the damage—referred to as the damage scattering signals.

By analyzing the first arrival time of the damage scattering signals and the propagation speed of the stress waves in the structure, the localization of the structural damage is determined. This damage localization method relies solely on the analysis of the structure’s response signals, without imposing any constraints on the structural characteristics. This means that the method is versatile and can be applied to detect a wide variety of concrete structures, irrespective of their shape or form. Therefore, this approach holds great potential for practical engineering applications, offering a reliable and efficient solution for damage detection and localization in concrete structures.

### 2.1. Stress Wave Propagation Theory

Vibration is the phenomenon of energy propagation through a medium in the form of waves. The vibration of particles transfers energy to neighboring particles, causing them to vibrate as well. Based on the nature of the vibration, stress waves can be classified into P-waves [27], S-waves [28], and surface waves [29]. In an ideal homogeneous medium, stress waves propagate in a straight line without any reflection or refraction. However, when stress waves travel from one medium to another, phenomena such as reflection and refraction occur at the interface between the two media [30,31]. When stress waves are reflected at the interface, the reflected waves are termed “reflected waves”, while the waves that pass through the interface are called “transmitted waves”. If the propagation angle changes after the wave crosses the interface, the resulting wave is referred to as a “refracted wave” [32].

When stress waves propagate through solid media, energy loss, known as energy attenuation, occurs. Depending on the mechanism responsible for the energy loss, stress wave attenuation in concrete can be categorized into three types: diffusion attenuation, absorption attenuation, and scattering attenuation. As stress waves propagate through concrete, the wavefronts continually expand, causing a decrease in sound pressure on the wavefront, which is known as diffusion attenuation. Absorption attenuation refers to the energy dissipation in the form of heat due to the viscous absorption and thermal conduction effects of the medium, resulting in a decrease in stress wave energy. Due to the heterogeneous nature of concrete, stress waves scatter as they propagate, causing the path of the scattered waves to become more complex. The dissipation of energy in the form of heat due to scattering is called scattering attenuation [33,34,35].

It should be noted that, although concrete is inherently a non-homogeneous material composed of aggregates, cement paste, and voids, this study adopts a simplified assumption of a homogeneous and isotropic medium to derive the theoretical wave propagation model. This effective medium approximation is widely used in wave-based SHM studies as it allows for tractable analysis and consistent estimation of wave velocities. The validity of this assumption is supported by experimental and numerical results presented in later sections, which demonstrate good agreement between theoretical predictions and observed data.

### 2.2. Wave Speed

In concrete SHM based on wave analysis, wave speed is a crucial evaluation index. Due to the propagation characteristics of waves, the propagation speed of the same type of wave in the same medium should be identical. Based on the wave equation for solid media [36], the propagation speed of P-waves, *C_LT_*, in one dimension is given by

(1) $$C_{LT}=\sqrt{\frac{E\left(1-\nu \right)}{\rho \left(1+\nu \right)\left(1-2\nu \right)}}$$

Propagation speed of S-waves, *C_i_*, is given by

(2) $$C_{i}=\sqrt{\frac{E}{2\rho \left(1+\nu \right)}}$$

Propagation speed of surface waves, *C_R_*, is given by

(3) $$C_{R}=\frac{0.87+1.12\nu }{1+\nu }\sqrt{\frac{E}{2\rho \left(1+\nu \right)}}$$

where *E* represents the elastic modulus of the propagation medium; *ν* represents Poisson’s ratio; *ρ* represents the density of the propagation medium.

A comparative analysis of the above three wave speed formulas reveals that, within the same medium, P-waves have the fastest speed, followed by S-waves and then surface waves. Therefore, in concrete structural damage detection, the first waves to be received are P-waves. Consequently, in most cases, the stress wave propagation speed in concrete structures, denoted as *ν_g_*, is considered to be the speed of P-waves, *C_LT_*.

**Figure 1:**
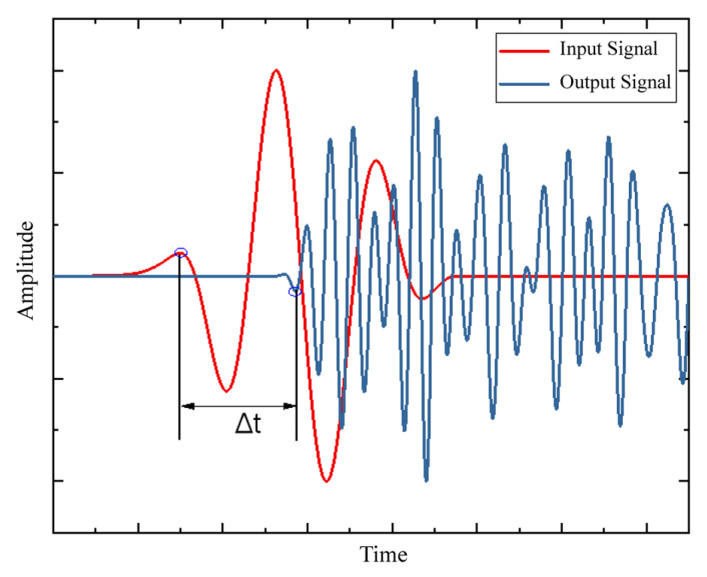
P-wave speed calculation.

In practical engineering applications, the elastic modulus and Poisson’s ratio of structural components may differ from those of standard concrete, and obtaining the actual values of these parameters can be complex. Therefore, the P-wave speed calculated using Equation (1) may differ from the actual wave speed in the specimen. In practical operations, the wave speed can be calculated based on the time difference between the output and input signals, as shown in Figure 1.

That is,

(4) $$v_{g}=\frac{\Delta x}{\Delta t}$$

where Δ*x* is the distance between the actuator and the sensor.

### 2.3. Damage Scattering Signals

The wave-based method for structural health monitoring relies on the installation of at least one pair of piezoelectric sensors on the structure. A voltage signal is applied to one sensor (the actuator), which generates a stress wave through the inverse piezoelectric effect [37]. This wave propagates through the concrete medium and is detected by another sensor (the receiver), which converts the mechanical deformation into an electrical signal via the direct piezoelectric effect. The resulting voltage signal constitutes the system’s response. To detect damage, the response signal under the damaged condition is compared to that under the healthy state. Any discrepancy between the two can be attributed to structural changes, primarily the presence of damage. These discrepancies form the damage scattering signal, which contains information about the damage’s location, size, and nature.

**Figure 2:**
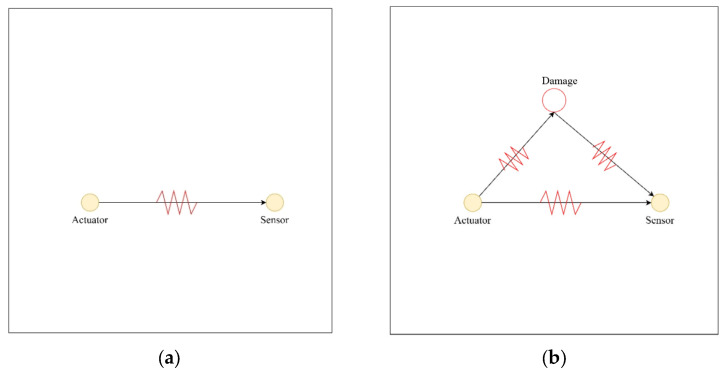
Schematic diagram of stress wave propagation of (**a**) healthy state and (**b**) damaged state.

**Figure 3:**
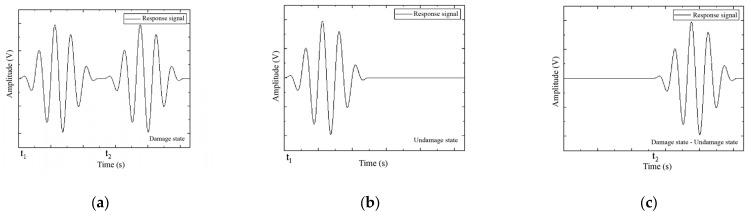
Damage scattering signal acquisition is obtained by (**a**) damaged response signal minus (**b**) undamaged response signal, as shown in (**c**) damage scattering signal.

As illustrated in Figure 2, when the structure is undamaged, the wave travels directly from actuator to receiver. When damage is present, part of the wave is scattered at the damage site and reflected toward the receiver, generating additional signal components. Assuming that other variables remain constant, the damage scattering signal can be obtained by baseline subtraction:

(5) $$Y_{d}\left(t\right)=Y_{ijb}\left(t\right)-Y_{ij}\left(t\right)$$

where *Y_ijb_*(*t*) and *Y_ij_*(*t*) represent the response signals in the damaged and healthy states, respectively, as shown in Figure 3. The damage scattering signal consists of the signal components that pass through the structure, scatter due to the damage, and arrive at the receiver. The damage scattering signal contains information about the location and extent of the damage. By performing feature analysis on the damage scattering signal, structural damage can be identified and localized.

Although the measured signals may contain some level of noise, this study focuses on the theoretical validation of the method and therefore does not explicitly model noise effects. The baseline subtraction is assumed to isolate damage-induced scattering, and the signal difference is treated as purely resulting from structural damage.

### 2.4. Concrete Damage Localization Method

In a single damage monitoring area, for the pair of actuators and sensors, if damage exists at a point *r_d_* (*x_d_*, *y_d_*), the distance at which the damage scattering signal reaches the receiver, *D_idj_*, is the sum of the distances from the actuator to the damage, *D_i_*, and from the damage to the receiver, *D_j_*:

(6) $$D_{idj}=D_{i}+D_{j}$$

As mentioned above, when the stress wave speed in a concrete structure is constant, the distance at which the damage scattering signal reaches the receiver, *D_idj_*, can be expressed as

(7) $$D_{idj}={\;v_{g}\times t_{idj}=D}_{i}+D_{j}$$

**Figure 4:**
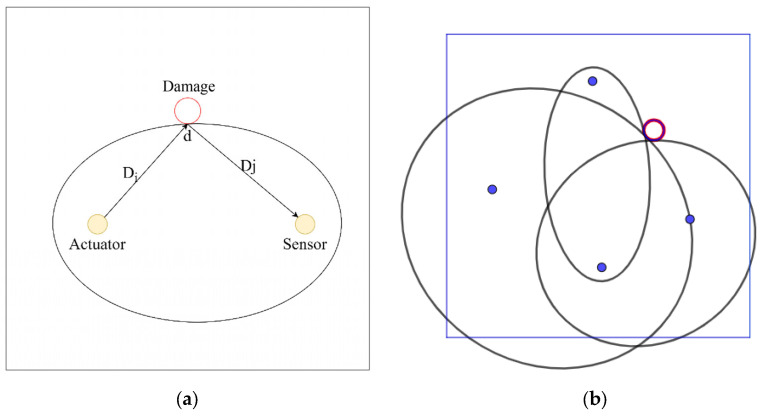
Schematic diagram of (**a**) damage reflection and (**b**) localization. The red circle represents the damage and the blue dots represent the positions of the SA (actuator and sensor pairs).

Since both *v_g_* and *t_idj_* are constants, it can be concluded that the result, *D_idj_*, is also a constant. Therefore, the damage reflection point is located on an ellipse with the actuator and sensor as the foci and the distance between the actuator and sensor, *D_idj_*, as the major axis, as shown in Figure 4a. Since *v_g_* is a constant, the distance from the actuator to the damage reflection point, *D_i_* + *D_j_*, is the shortest distance, meaning the signal reflected from the damage point is the first to be received by the sensor. Therefore, the first arrival signal in the damage scattering signal is the signal reflected by the damage reflection point. The propagation time can be calculated based on the time difference between the start of the damage scattering signal and the excitation signal, as shown below:

(8) $$t_{idj}=t_{2}-t_{j}$$

where *t*_2_ and *t_j_* are the start times of the damage scattering signal and the excitation signal, respectively.

For concrete damage, circular damage can encompass most damage types. By determining three different boundary reflection points, a circle can be established to localize concrete hole damage. SA can serve as both actuators and receivers. Therefore, only three SA are needed to identify and localize damage. However, when the damage is located along the line connecting two sensors, there is a significant error when applying the damage scattering signal-based method for concrete damage localization, and a boundary reflection point cannot be determined. To ensure accurate localization of concrete hole damage, four SA are arranged. Three different ellipses are obtained from the first wave arrival times of three sets of damage scattering signals and the stress wave speed, and the outer circle of the three ellipses is determined to localize the damage. Once enough reflection points are determined, the basic outline of the damage can be established, enabling localization and quantification of the damage, as shown in Figure 4b.

## 3. Experiments and Results

### 3.1. Experimental Specimens

**Figure 5:**
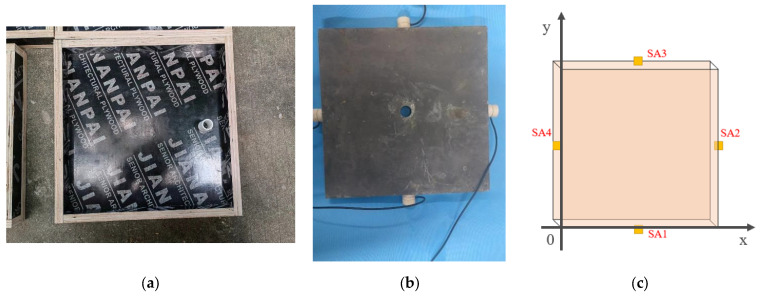
Specimen of (**a**) template, (**b**) post-pouring and (**c**) smart aggregates (SA) are located at the center of each of the edge.

To simulate the internal damage conditions of concrete structures, seven total concrete specimens with dimensions of 300 mm × 300 mm × 30 mm were designed. By pre-setting holes in the specimens, internal discontinuities in the medium were created to simulate internal damage in the concrete structure. The concrete used in this experiment was C30 concrete. To simulate internal defects in the structure, PVC pipes were fixed in the mold, and, after the concrete solidified, the PVC pipes were removed. The pictures of the specimen are shown in Figure 5, while the specific parameters are shown in Table 1.

The specimen numbers S0, S20, and S30 represent the healthy state, damage with a 20 mm diameter, and damage with a 30 mm diameter, respectively. L1, L2, and L3 represent the damage center positions at (150 mm, 150 mm), (205 mm, 205 mm), and (205 mm, 150 mm), with the coordinate axes originating from the bottom-left corner of the specimen, as shown in Figure 5c. The smart aggregates (SA) were attached at the center of each of the four edges.

It is acknowledged that, in practical scenarios, concrete damage most commonly manifests as cracks with well-defined geometry. In this study, circular holes were adopted in the experimental design due to their ease of fabrication and control, enabling consistent evaluation of the damage localization method. These voids can be considered simplified representations of localized damage or early-stage crack formation.

### 3.2. Experimental Setup

**Figure 6:**
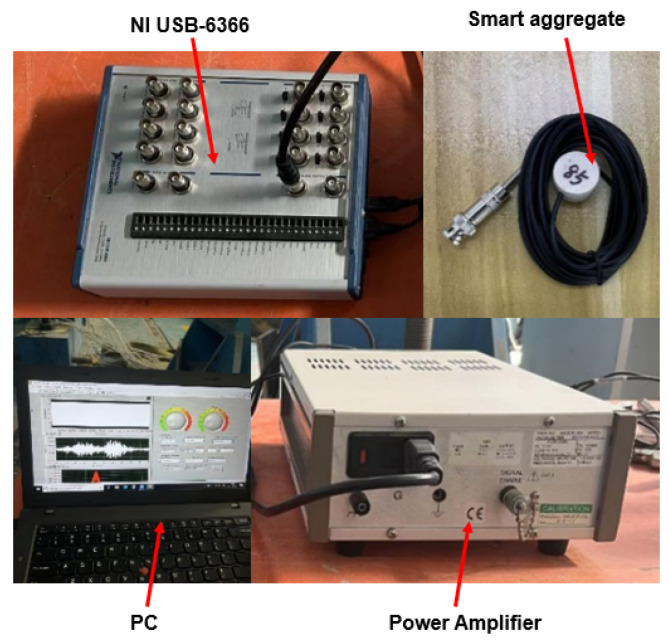
Experimental monitoring setup.

The experimental setup includes Smart Aggregates (SA), signal amplifiers, NI USB-6366 data acquisition cards, and computers, as shown in Figure 6. The SA used were produced by Qinhuangdao Hengke Technology Co., Ltd. (Qinhuangdao, China). The NI USB-6366 data acquisition card, made by National Instruments (NI, Austin, TX, USA), is a synchronous sampling multifunctional data acquisition device with 8 input channels and a maximum sampling rate of 2 MS/s. By combining the NI USB-6366 with LabVIEW 2023, specific signal forms can be generated, and signal reception can be achieved.

**Figure 7:**
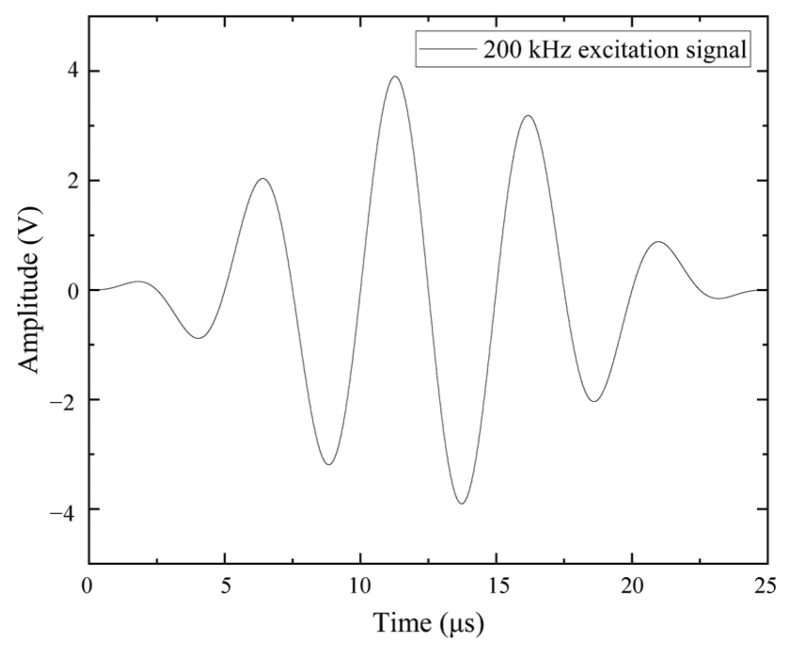
Excitation signal waveform.

Generally, the higher the frequency of the detected signal, the better the spatial resolution. However, this also leads to greater attenuation. Considering that the specimens in this experiment are relatively small, to achieve better spatial resolution, a Gaussian-modulated sine wave signal with a central frequency of 200 kHz was selected as the excitation signal. The specific expression is given by Equation (9), with an amplitude of 4 V and a central frequency, *f*, of 200 kHz. The waveform is shown in Figure 7. The sampling frequency during the experiment was 1 MS/s.

(9) $$I\left(t\right)=(sign(t)-sign(t-5/f))\times (1-cos(0.4\times \pi \times f\times t))\times sin(2\times \pi \times f\times t)$$

Firstly, data collection was performed on specimen S0 to obtain the response signals between the sensors under healthy conditions. These response signals will serve as the baseline for subsequent damage identification. Data collection was then performed under other conditions for the sensors. In this experiment, the active propagation method based on SA was applied, with SA externally attached to the specimen, serving as both actuators and receivers. A voltage signal was applied to the actuator, and, due to the inverse piezoelectric effect, a stress wave was generated and propagated through the medium, then received by the receiver. When the structure suffers damage, an inhomogeneous region is created within the medium, and when the stress wave propagates to the interface of this inhomogeneous region, reflection occurs. The reflected wave is received by the receiver. It is assumed that the difference between the response signals in the healthy and damaged states is caused solely by the damage. Therefore, the damage scattering signal can be obtained through baseline subtraction. The localization and identification of the damage can then be achieved by analyzing the arrival time of the first wave of the damage scattering signal and the propagation speed of the stress wave.

### 3.3. Data Processing and Results Analysis

#### 3.3.1. Wave Speed Calculation

**Figure 8:**
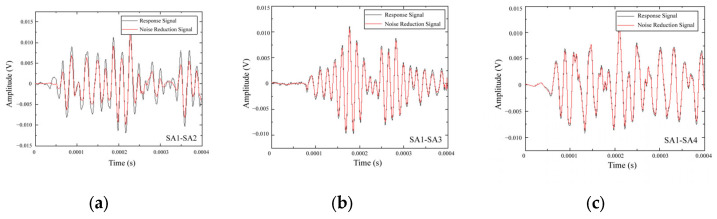
Response signals for specimen S0 of (**a**) SA1-SA2, (**b**) SA1-SA3, and (**c**) SA1-SA4 sensor pairs.

The signal used in this experiment is a Gaussian-modulated sine wave with a central frequency of 200 kHz. The time delay of the Gaussian-modulated signal is 1.8 μs. The S0 specimen was used for wave speed analysis, as it represents a healthy state concrete specimen, meaning that the stress wave propagation is not affected by any damage. The actuator SA1 and the remaining three sensors were used as receivers for wave propagation analysis. The center-to-center distances from the actuator SA1 to the receivers SA2, SA3, and SA4 were 212 mm, 300 mm, and 212 mm, respectively. By collecting the voltage changes at the receivers SA2, SA3, and SA4, the time-domain voltage signals of the sensors were obtained. The voltage response signals were denoised using a three-level wavelet transform, as shown in Figure 8.

The Daubechies-4 (db4) wavelet was selected as the basis function due to its compact support and suitability for transient signal analysis. A soft-thresholding scheme was used to suppress noise components in the detail coefficients, and the denoised signal was reconstructed via inverse wavelet transform. This approach has proven effective in processing ultrasonic and stress wave signals in structural health monitoring [38,39].

The first wave signals collected by SA in concrete structures are usually P-waves. According to the design specifications for concrete structures, the elastic modulus of C30 concrete is 30 GPa, and Poisson’s ratio is 0.25. Based on the wave equation for solid media (Equation (1)), the P-wave speed for standard C30 concrete should be 3832 m/s.

When using Equation (4) to calculate the P-wave speed, the results were summarized in Table 2. The calculated wave speed in the concrete specimens used in this experiment is 3847 m/s, and the maximum deviation from the average stress wave speed is no more than 5%. Comparing the wave speeds calculated using Equations (1) and (4), the values are nearly identical. Therefore, in the experiment, the P-wave speed can be calculated based on the first wave arrival time, and this method does not require the calculation of the elastic modulus and Poisson’s ratio, significantly optimizing the difficulty of wave speed determination. Thus, the average wave speed of 3847 m/s, calculated using Equation (4), was adopted as the stress wave speed for the concrete specimens used in this study.

#### 3.3.2. Damage Scattering Signal

**Figure 9:**
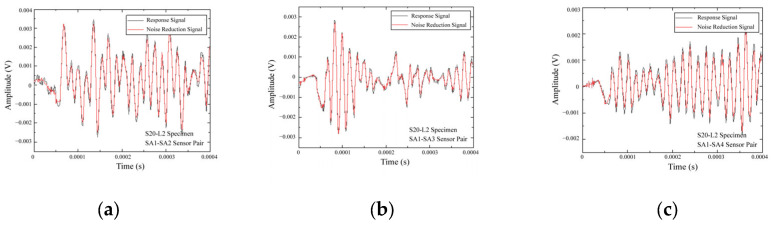
Response signals for specimen S20-L2 of (**a**) SA1-SA2, (**b**) SA1-SA3, and (**c**) SA1-SA4 sensor pairs.

**Figure 10:**
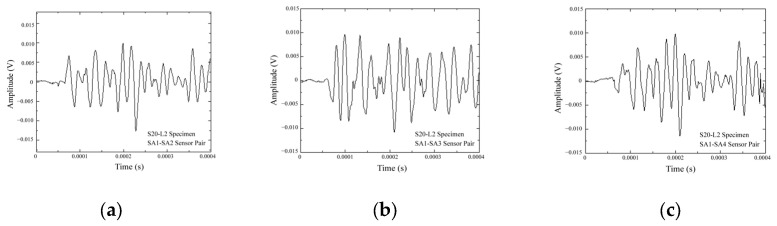
Damage scattering signals for specimen S20-L2 of (**a**) SA1-SA2, (**b**) SA1-SA3, and (**c**) SA1-SA4 sensor pairs.

Assuming that the difference between the response signals in the healthy and damaged states is solely caused by the damage within the structure. The damage scattering signal can be obtained through baseline subtraction, as shown in Equation (5). The damage scattering signal is obtained by subtracting the response signal under the healthy state from the response signal under the damaged state. Figure 9 shows the response signals under various damage conditions while Figure 10 shows the damage scattering signals (For the sake of brevity, only the response signal and damage scattering signal of specimen S20-L2 is shown). From the first arrival time of the damage scattering signal, the propagation time of the damage scattering wave can be determined, and the propagation times of the first waves for the damage scattering signals are shown in Table 3.

#### 3.3.3. Damage Identification and Localization

**Figure 11:**
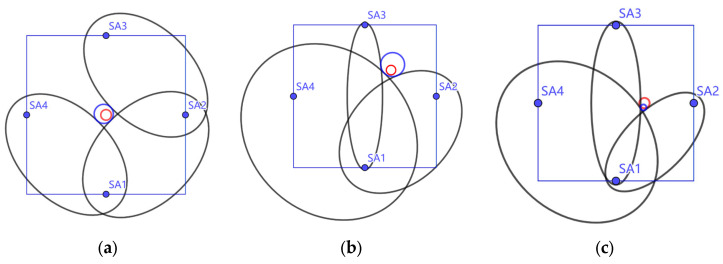

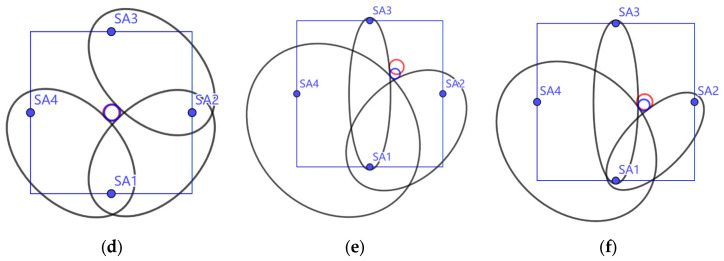
Localization analysis results for specimen (**a**) S20-L1, (**b**) S20-L2, (**c**) S20-L3, (**d**) S30-L1, (**e**) S30-L2, and (**f**) S30-L3. The square represents the specimen boundary, with the four blue points indicating the positions of sensors SA1, SA2, SA3, and SA4. The red circle represents the real damage location in the specimen, while the blue circle represents the estimated localization result from the experiments.

Based on the wave speed and the first arrival time of the damage scattering signal, as well as the positional relationships between the sensor pairs, an ellipse can be obtained, and one boundary point of the damage will lie on this ellipse. A circle can encompass most shapes, and when three boundary points of a circle are found, the shape can be determined. Therefore, to locate the damage, three different ellipses need to be obtained, and the outer circle of these three ellipses will provide an approximation of the damage location. Based on the principles of the damage localization method described earlier, damage localization was analyzed for each specimen, and the results are shown in Figure 11.

From the localization results, it can be seen that the method of determining the damage location based on the wave speed and the damage scattering signal is effective, with errors falling within acceptable limits. The maximum deviation for the damage center position is 14.2 mm, and the maximum error for the damage size is 14.5 mm. A detailed comparison of the results is provided in Table 4. The experimental results show that this method can effectively identify and localize concrete damage, with good identification accuracy. The maximum localization error observed in the experiments was 14.5 mm, which is less than 5% of the specimen size and within one damage diameter. This level of accuracy is considered acceptable for preliminary damage screening in practical applications. It is also noteworthy that the average localization error was lower, and finite element simulations under ideal conditions yielded even smaller errors, demonstrating the potential of the method for reliable localization with further refinement.

#### 3.3.4. Uncertainty Analysis

The accuracy of the proposed damage localization method is influenced by multiple sources of uncertainty, including wave speed estimation, signal timing precision, and structural material heterogeneity.

The wave speed was calculated based on multiple sensor paths, and the standard deviation among paths was less than 2.2%, suggesting relatively stable propagation conditions. The data acquisition system operated at a sampling rate of 1 MS/s, implying a timing resolution of 1 μs. Given an average wave speed of 3847 m/s, this introduces a theoretical spatial uncertainty of ±3.8 mm for each signal timing measurement.

Accumulated across two paths (actuator-to-damage and damage-to-sensor), this could yield an elliptical localization uncertainty of ~6 to 8 mm, depending on damage geometry and location. Overall, the maximum observed localization error in this study was 14.5 mm, which is within acceptable margins for structural monitoring applications.

## 4. Numerical Simulation

The COMSOL 6.0 finite element analysis software is used to build models of concrete structures and piezoelectric ceramic sensors, simulating the electro-mechanical multiphysics coupling between the concrete structure and the SA. The simulation allows for the excitation and reception of stress wave signals within the concrete structure. Various damage scenarios with different sizes and locations of defects are designed in the concrete structure. The damage scattering signals received from the sensors are analyzed to localize the damage.

### 4.1. Numerical Model

#### 4.1.1. Specimen

The creation of the model is the foundation of the finite element analysis. Since this study primarily investigates stress wave propagation and reflection in a plane, and to improve the finite element calculation efficiency, the finite element model uses plane strain elements.

The model was constructed using COMSOL finite element software for both the concrete slab and the SA, with material properties assigned to each. The SA were affixed to the center of each side of the concrete slab, with their polarization direction aligned in the 3-direction. The concrete slab dimensions are 300 mm × 300 mm, and a damage was introduced into the slab, as shown in Table 1. The coordinate system is set with the origin at the bottom-left corner of the slab, as shown in Figure 5c.

#### 4.1.2. Material Parameters

During the study, the concrete slab remained in an elastic stress state, so only its Young’s modulus, *E*, Poisson’s ratio, *μ*, and density, *ρ*, need to be defined. In this study, the concrete is assumed to be a homogeneous elastic medium with Rayleigh damping. Thus, a damping coefficient is defined for the simulation. The specific material parameters for concrete are listed in Table 5.

The SA made by lead zirconate titanate (PZT = PbZr_1−x_Ti_x_O_3_) is anisotropic. Therefore, when defining its material properties, the polarization direction of the piezoelectric ceramic must be specified. The direction perpendicular to the long side of the PZT, pointing upwards, is defined as the polarization direction (the 3-direction). In this study, the piezoelectric material properties also need to define its density, elastic matrix, relative permittivity matrix, and coupling matrix. The specific parameters for PZT are shown in Table 6. The PZT parameters used in the simulation were based on the standard properties of PZT-5A, as provided in the COMSOL Multiphysics^®^ material library.

#### 4.1.3. Boundary

When stress waves propagate through the concrete medium, reflection and diffraction occur when the waves reach the boundaries of the specimen. These behaviors lead to reflected waves, which are also received by the piezoelectric ceramic sensors. In actual engineering, the concrete structure has finite dimensions, and boundary reflections cannot be ignored. Moreover, the relatively small size of the experimental specimens amplifies the boundary reflection effects. Therefore, to accurately simulate the stress wave propagation process, elastic boundary conditions are used in this study. This means that, when stress waves reach the specimen boundaries, they are reflected back.

#### 4.1.4. Mesh Division

**Figure 12:**
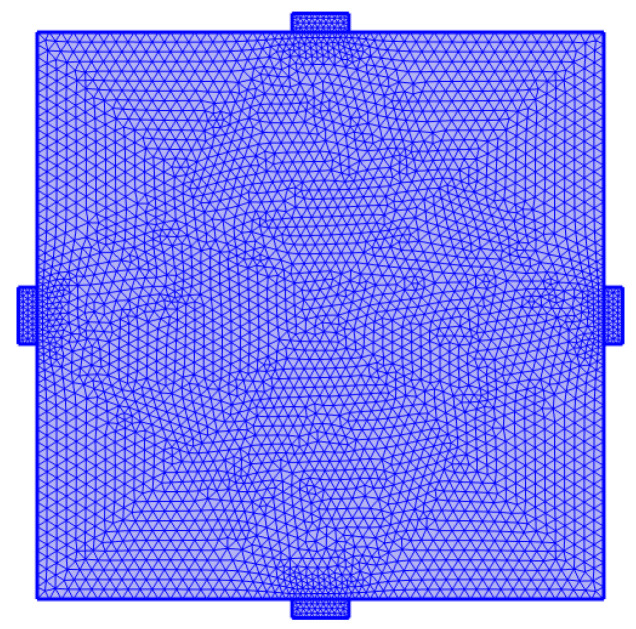
Mesh division.

If the mesh is too fine, the calculation time will be significantly extended. Conversely, if the mesh is too coarse, the accuracy of the simulation results will be affected. In this simulation, an extremely fine mesh size was used, with the maximum element size set to 3.2 mm and the minimum element size set to 0.0064 mm. The mesh shape chosen was free triangular elements. The mesh division results are shown in Figure 12.

#### 4.1.5. Data Acquisition

For this simulation, the excitation signal selected was a Gaussian-modulated sine wave with a central frequency of 200 kHz, an amplitude of 0.94 V, and a time delay of 1.79 μs. The modulation formula is shown in Equation (10). To save computation time and ensure the accuracy of the results, the simulation was set to run for 150 time steps.

(10) $$f=\left(1-cos\left(2\times \pi \times f_{0}\times \frac{t}{3}\right)\right)\times sin\left(2\times \pi \times f_{0}\times t\right)$$

where *f*_0_ is the central frequency of the signal.

### 4.2. Data Processing and Results Analysis

#### 4.2.1. Stress Wave Propagation Process

**Figure 13:**
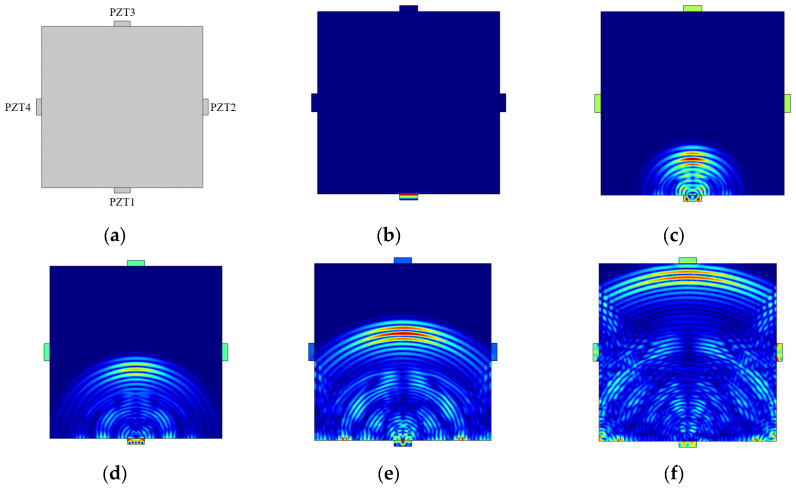
(**a**) Model and piezoelectric arrangement; stress wave at the (**b**) initial moment, *t* = 0, (**c**) *t* = 26 μs, (**d**) *t* = 42 μs, (**e**) *t* = 58 μs, and (**f**) *t* = 82 μs.

The model dimensions and piezoelectric arrangement are shown in Figure 13a. Stress wave propagation in the concrete slab under healthy conditions is analyzed and studied, as shown in Figure 13b. At the starting moment, an electric field is applied to the piezoelectric ceramic, causing it to deform due to the inverse piezoelectric effect. The concrete slab generates stress waves under the excitation, which propagate in a spherical shape within the concrete medium. As shown in Figure 13c, at a certain time *t* = 26 μs, the stress wave clearly exhibits spherical propagation. The stress waves in the vertical polarization direction are shown in red, while the waves on either side are in lighter colors, indicating that the energy in the vertical direction is larger. At a later time (*t* = 42 μs), as shown in Figure 13d, the stress wave propagates to the boundary, where reflection occurs. The reflected wave and the original stress wave partially cancel each other out, resulting in a decrease in the intensity of the original stress wave. As shown in Figure 13e, at time *t* = 58 μs, the first wave of the stress wave reaches PZT2 and PZT4, meaning that the stress wave signal is received by the PZT sensors. Simultaneously, the color of the piezoelectric ceramics gradually darkens, and they undergo strain. Due to the direct piezoelectric effect, an electrical effect is generated on the SA, which leads to a voltage signal change that is collected. At a later time (*t* = 82 μs), as shown in Figure 13f, the first wave of the stress wave reaches PZT3, and the stress wave signal is received by PZT3.

#### 4.2.2. Stress Wave Speed Analysis

Using PZT1 as the actuator and the remaining three sensors as receivers, the wave propagation is analyzed using the wave-based method. The center-to-center distances from the actuator PZT1 to the receivers PZT2, PZT3, and PZT4 are 212 mm, 300 mm, and 212 mm, respectively. By collecting the voltage variations at the receivers PZT2, PZT3, and PZT4, the time-domain voltage signals of the sensors are obtained. The stress wave propagation speed in the concrete slab is calculated based on the time difference between the excitation signal and the received signal. The specific parameters are shown in Table 7. From the table, the calculated wave speeds are 3833 m/s, 3744 m/s, and 3833 m/s. The speed error is less than 3%, so the average wave speed of 3803 m/s is used as the group speed of the stress wave.

#### 4.2.3. Damage Scattering Signal Analysis

By subtracting the response signal under healthy conditions from the response signal under damaged conditions, the damage scattering signal of the structure is obtained. Using the damage scattering signal, the propagation time can be used to localize the damage. The damage scattering signals for the above specimens were analyzed, and the arrival times of the signals are shown in Table 8.

#### 4.2.4. Localization Results

**Figure 14:**
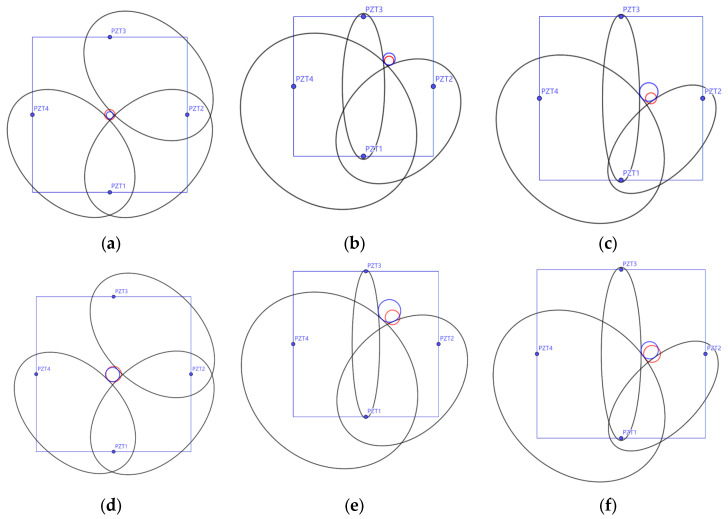
Localization analysis result for (**a**) S20-L1, (**b**) S20-L2, (**c**) S20-L3, (**d**) S30-L1, (**e**) S30-L2, and (**f**) S30-L3. The square represents the specimen boundary, with the four points indicating the positions of sensors PZT1, PZT2, PZT3, and PZT4. The red circle represents the real damage location in the specimen, while the blue circle represents the estimated localization result from the numerical simulation.

By using the stress wave speed and signal propagation times, ellipses are obtained with the sensor pairs as the foci. By finding the outer tangent of three ellipses, the damage location can be determined, as shown in Figure 14.

From the localization results, it can be concluded that the method based on stress wave speed and damage scattering signals is effective in determining the damage location. The errors are within acceptable limits, with the maximum deviation for the damage center position being 12.5 mm and the maximum error for the damage size being 8.3 mm. The detailed comparison of the results is shown in Table 9.

In engineering applications, SA can be arranged on the surface or inside concrete structures. By using the stress wave speed and the first wave arrival time of the damage scattering wave, the geometric location of the damage can be obtained. The localization and identification of damage using the damage scattering signal and wave speed do not require the destruction of the concrete structure, and the method provides high measurement accuracy with minimal error, fewer sensors, and simple operation. This allows for quick and effective localization of the damage.

## 5. Conclusions and Discussion

In this study, an active piezoelectric monitoring technique was employed to propose a damage identification and localization method for concrete based on damage scattering signals. This method uses response signals collected under healthy conditions as the baseline. When structural damage occurs, corresponding response signals are collected using the wave-based method. By comparing the response signals in the damaged state with those in the healthy state, damage scattering signals caused by structural damage are obtained. By analyzing the first arrival time of the damage scattering signal, the geometric relationship between the structural damage and the sensor pair can be established, enabling the localization of the damage. A theoretical analysis method for damage identification and localization based on damage scattering signals was established for concrete hole damage.

In the experiments, a concrete slab with pre-set holes was used as a specimen to verify the correctness and reliability of the proposed damage identification and localization method. A specific excitation signal was applied to the actuator using LabVIEW 2023, and response signals from other sensors were collected. The method was then applied for processing and analysis, and the results confirmed that the method is feasible for damage at different locations, as well as for damage diameters of 20 mm and 30 mm. The errors in the analysis were mainly attributed to noise effects and limitations in the sampling frequency.

What is more, a parametric analysis based on the stress wave propagation model was conducted to achieve the identification, localization, and quantitative assessment of concrete damage. Seven concrete slabs were established, and each specimen was analyzed using the wave-based method. The stress wave propagation within the specimens was observed, validating the rationality of the method. The data from the collected response signals were processed and analyzed, and the results effectively localized the damage. The simulation results were corroborated by the experimental results, proving the accuracy of the numerical simulation method.

The main innovations of this study are as follows: Development of a concrete damage identification and localization method based on damage scattering signals. By comparing the response signals under damaged and healthy conditions, scattering signals caused by structural damage are obtained. The first arrival time of the damage scattering signals is analyzed to determine the geometric relationship between the structural damage and the sensor pair, thereby localizing the damage. This method analyzes the response signals in the time domain, reducing the complexity of data analysis and saving significant analysis time, making the localization of concrete damage more efficient. Development of a damage localization method based on damage scattering signals and stress wave speed. This method is an analysis-based approach using response signals, which does not rely on structural models, making it more suitable for practical engineering applications.

The number and placement of Smart Aggregates (SAs) have a direct impact on the damage localization accuracy. In this study, four sensors were deployed symmetrically along the edges of the specimen to ensure sufficient coverage and redundancy. Our results indicate that using fewer than four sensors may lead to insufficient ellipse intersections for accurate triangulation, particularly if the damage lies directly between two sensors. In such cases, the scattered wave may not provide a distinguishable time-of-flight difference, resulting in larger localization errors. Finite element simulations further confirmed that asymmetric or colinear sensor placement leads to increased uncertainty. Conversely, spatially diverse configurations with at least three independent ellipse paths yielded the best performance, reducing localization error to within ~10 mm.

However, the method proposed in this study is designed for identifying damage in a single state of concrete, but it has limitations when applied to multi-damage states in concrete structures. Future research should address the capability of this method to handle multiple simultaneous damages. Furthermore, the noise issue was not thoroughly analyzed in this study. Further investigation is needed to understand the impact of noise on the localization method and to develop strategies to mitigate noise effects for more accurate results. What is more, this study used four SA to achieve damage identification and localization for single-state concrete damage. The optimization of sensor placement remains an area for further research, as efficient sensor configuration could improve the method’s effectiveness and reduce the number of sensors needed. Nevertheless, the results confirm that the method retains sufficient sensitivity to detect and localize multiple close-range defects, highlighting its potential for practical application in complex structural conditions.

It is acknowledged that environmental factors such as temperature fluctuations, varying moisture content, and internal stress states can significantly affect wave propagation characteristics and sensor performance in concrete structures. This study was conducted under standard laboratory conditions to isolate and verify the effectiveness of the proposed method. Future work will also focus on evaluating the robustness and adaptability of the method under variable environmental conditions, including temperature-compensated signal analysis and moisture-sensitive response calibration.

## Figures and Tables

**Table 1:** Specimen parameters.

Specimen Number	Length (mm)	Width (mm)	Damage Diameter (mm)	Damage Position (mm, mm)
S0	300	300	/	/
S20-L1	300	300	20	(150, 150)
S20-L2	300	300	20	(205, 205)
S20-L3	300	300	20	(205, 150)
S30-L1	300	300	30	(150, 150)
S30-L2	300	300	30	(205, 205)
S30-L3	300	300	30	(205, 150)

**Table 2:** Experimental wave speed calculation.

Sensor Pair	Distance (mm)	Δ*t* (μs)	Speed (m/s)	Average Speed (m/s)
SA1-SA2	212	54.2	3911	3847
SA1-SA3	300	79.2	3788	
SA1-SA4	212	55.2	3841	

**Table 3:** Signal propagation time.

Specimen	Sensor Pair	First Wave Arrival Time (μs)
S20-L1	SA1-SA2	75
	SA1-SA4	73
	SA2-SA3	74
S20-L2	SA1-SA2	79
	SA1-SA3	82
	SA1-SA4	106
S20-L3	SA1-SA2	65
	SA1-SA3	85
	SA1-SA4	91
S30-L1	SA1-SA2	75
	SA1-SA4	74
	SA2-SA3	74
S30-L2	SA1-SA2	77
	SA1-SA3	83
	SA1-SA4	103
S30-L3	SA1-SA2	64
	SA1-SA3	83
	SA1-SA4	90

**Table 4:** Comparison of the estimated results with the real damage.

Specimen Number	Real Damage Location (mm, mm)	Estimated Damage Location (mm, mm)	Real Damage Radius (mm)	Estimated Damage Radius (mm)
S20-L1	(150, 150)	(145.7, 152.0)	10.0	18.0
S20-L2	(205, 205)	(208.0, 217.0)	10.0	24.5
S20-L3	(205, 150)	(203.0, 141.6)	10.0	5.8
S30-L1	(150, 150)	(151.7, 150.1)	15.0	15.0
S30-L2	(205, 205)	(201.7, 191.2)	15.0	10.5
S30-L3	(205, 150)	(203.6, 144.0)	15.0	11.0

**Table 5:** Parameters of concrete.

Material	Young’s Modulus (GPa)	Density (kg/m^3^)	Poisson’s Ratio	Damping Ratio
Concrete	30	2500	0.25	0.05

**Table 6:** Parameters of PZT.

Material	Density, *ρ* (kg/m^3^)	Relative Permittivity Matrix	Elastic Matrix (Pa)	Coupling Matrix
PZT	7500	$\varepsilon_{11}=762.5$ $\varepsilon_{22}=762.5$ $\varepsilon_{33}=663.2$	$C_{11}=1.38999\;\times \;10^{11}$ $C_{12}=7.78366\;\times \;10^{10}$ $C_{22}=1.38999\;\times \;10^{11}$ $C_{13}=7.42836\;\times \;10^{10}$ $C_{23}=7.42836\;\times \;10^{10}$ $C_{33}=1.15412\;\times \;10^{11}$ $C_{44}=2.5641\;\times \;10^{10}$ $C_{55}=2.5641\;\times \;10^{10}$ $C_{66}=3.0581\;\times \;10^{10}$ (Values not specified are assumed to be 0)	$E_{31}=-5.20279$ $E_{32}=-5.20279$ $E_{33}=15.0804$ $E_{24}=12.7179$ $E_{15}=12.7179$ (Values not specified are assumed to be 0)

**Table 7:** Numerical wave speed calculation.

Sensor Pair	Distance Between Sensor Pairs (mm)	Δ*t* (μs)	Speed (m/s)	Average Speed (m/s)
PZT1-PZT2	212	55.31	3833	3803
PZT1-PZT3	300	80.11	3744	
PZT1-PZT4	212	55.31	3833	

**Table 8:** Signal Arrival Times.

Specimen	Sensor Pair	First Wave Arrival Time (μs)
S20-L1	PZT1-PZT2	78.1
	PZT1-PZT4	78.2
	PZT2-PZT3	78.7
S20-L2	PZT1-PZT2	81.9
	PZT1-PZT3	83.6
	PZT1-PZT4	108.1
S20-L3	PZT1-PZT2	67.3
	PZT1-PZT3	82.2
	PZT1-PZT4	91.6
S30-L1	PZT1-PZT2	76.2
	PZT1-PZT4	75.0
	PZT2-PZT3	76.5
S30-L2	PZT1-PZT2	83.0
	PZT1-PZT3	81.9
	PZT1-PZT4	105.3
S30-L3	PZT1-PZT2	68.2
	PZT1-PZT3	81.9
	PZT1-PZT4	91.9

**Table 9:** Comparison of results.

Specimen Number	Real Damage Location (mm, mm)	Estimated Damage Location (mm, mm)	Real Damage Radius (mm)	Estimated Damage Radius (mm)
S20-L1	(150, 150)	(149.9, 148.9)	10.0	6.9
S20-L2	(205, 205)	(205.1, 208.8)	10.0	12.9
S20-L3	(205, 150)	(200.1, 161.5)	10.0	16.9
S30-L1	(150, 150)	(148.0, 149.1)	15.0	13.7
S30-L2	(205, 205)	(198.7, 218.4)	15.0	23.3
S30-L3	(205, 150)	(200.7, 156.5)	15.0	15.3

## Data Availability

The data are available from the corresponding author on reasonable request.

## Abbreviations

The following abbreviations are used in this manuscript:

SHM	structural health monitoring
NDT	non-destructive testing
SA	smart aggregates
